# Making sense of hormone-mediated defense networking: from rice to *Arabidopsis*

**DOI:** 10.3389/fpls.2014.00611

**Published:** 2014-11-11

**Authors:** David De Vleesschauwer, Jing Xu, Monica Höfte

**Affiliations:** Laboratory of Phytopathology, Department of Crop Protection, Faculty of Bioscience Engineering, Ghent UniversityGhent, Belgium

**Keywords:** hormone signaling, plant immunity, *Oryza sativa*, plant defense, disease resistance, microbial virulence, pathogen

## Abstract

Phytohormones are not only essential for plant growth and development but also play central roles in triggering the plant immune signaling network. Historically, research aimed at elucidating the defense-associated role of hormones has tended to focus on the use of experimentally tractable dicot plants such as *Arabidopsis thaliana*. Emerging from these studies is a picture whereby complex crosstalk and induced hormonal changes mold plant health and disease, with outcomes largely dependent on the lifestyle and infection strategy of invading pathogens. However, recent studies in monocot plants are starting to provide additional important insights into the immune-regulatory roles of hormones, often revealing unique complexities. In this review, we address the latest discoveries dealing with hormone-mediated immunity in rice, one of the most important food crops and an excellent model for molecular genetic studies in monocots. Moreover, we highlight interactions between hormone signaling, rice defense and pathogen virulence, and discuss the differences and similarities with findings in *Arabidopsis*. Finally, we present a model for hormone defense networking in rice and describe how detailed knowledge of hormone crosstalk mechanisms can be used for engineering durable rice disease resistance.

## INTRODUCTION

Plant hormones are small signaling molecules that are essential in the regulation of plant growth, development, reproduction, and survival. They not only orchestrate intrinsic developmental programs but also convey environmental inputs and drive adaptive responses to a wide variety of biotic and abiotic stresses. Plants typically respond to pathogen infection or herbivore attack with a complex scenario of sequential, antagonistic, or synergistic action of different hormone signals leading to defense gene expression (Robert-Seilaniantz et al., 2011). This interplay or so-called crosstalk among individual hormone pathways enables plants to adjust their inducible defense arsenal to the type of attacker encountered and to use their limited resources in a cost-efficient manner (Pieterse et al., 2009, 2012).

Historically, plant hormone research has been polarized toward the use of the experimentally tractable dicot plant *Arabidopsis thaliana*. In this model species, the production and joint role of salicylic acid (SA), jasmonic acid (JA), and ethylene (ET) upon pathogen attack is well studied and these three hormones are considered to be the key players in the regulation of the disease signaling pathways. Following microbe perception, plants produce a complex blend of SA, JA, and ET, with the exact combination seemly depending on the infection strategy and lifestyle of the invading pathogen. Although there are exceptions, SA is usually effective against biotrophic pathogens that feed on living plant tissues, whereas cell death-provoking necrotrophic pathogens are commonly deterred by JA- and ET-dependent defenses (Bari and Jones, 2009). Moreover, these two pathways often interact in an antagonistic manner, which has led many authors to suggest that plant immunity follows a binary model with SA and JA/ET having opposite roles. In compliance with this concept, many other hormones, including abscisic acid (ABA), gibberellins (GAs), auxins and cytokinins (CKs), have been shown to differentially affect *Arabidopsis* resistance against biotrophs and necrotrophs by feeding into the SA-JA-ET cascades (Robert-Seilaniantz et al., 2007, 2011).

Although *Arabidopsis* has been an excellent model for studying hormone defense networking, recent studies using alternative model systems such as rice (*Oryza sativa* L.) are starting to provide important new insights, often revealing unique complexities (De Vleesschauwer et al., 2013; Yang et al., 2013). Consumed daily by more than 3 billion people worldwide and accounting for up to 50% of the daily caloric uptake of the world’s poor, rice is arguably the world’s most important staple food. Moreover, due to its relatively small and fully sequenced genome, its ease of transformation, accumulated wealth of genetic and molecular resources, and extensive synteny and collinearity with other cereals, rice has emerged as an excellent model for molecular genetic studies in monocots (Jung et al., 2008).

Here, we survey recent advances dealing with hormone-regulated defense networking in rice, focusing on interactions between hormone signaling, rice defense, and pathogen virulence. We will discuss the roles of the various hormone pathways in rice, paying special attention to the differences and similarities with findings in *Arabidopsis*. Finally, we describe how detailed knowledge of hormone defense networking can be used for engineering durable disease resistance in rice and outline some avenues for further research. For more detailed information on innate immune mechanisms and hormone biology in rice and other cereals, we refer the reader to a number of excellent recent reviews (Chen and Ronald, 2011; Liu et al., 2013a; Kyndt et al., 2014).

## PLANT IMMUNITY AND HORMONAL REGULATION

Plants live in complex environments where they are continuously subjected to attack by microbial pathogens and herbivorous insects. To combat infection by these deleterious organisms, plants possess a multilayered immune system that is composed of two interconnected branches, termed PAMP-triggered immunity (PTI) and effector-triggered immunity (ETI). PTI is triggered by perception of invariant pathogen- or microbe-associated molecular patterns (PAMPs/MAMPs). Several PAMPs have been identified thus far, including bacterial flagellin, lipopolysaccharides (LPSs) and elongation factor Tu (EF-Tu), fungal chitin and oomycetes cellulose-binding elicitor proteins (Schwessinger and Ronald, 2012). PAMPs are detected by means of high-affinity membrane-bound receptor proteins referred to as pattern recognition receptors (PRRs). Showing structural and functional similarities with animal Toll-like receptors, PRRs typically consist of an extracellular leucine-rich repeat (LRR) domain and an intracellular kinase domain (Zipfel, 2008). Perception of MAMPs by PRRs initiates a diverse set of downstream signaling events, leading to a basal level of resistance (Schwessinger and Ronald, 2012; Macho and Zipfel, 2014). In most cases, PTI is sufficient to impede pathogen colonization and suppress disease development. Successful pathogens, however, dodge PTI-based surveillance by delivering small effector proteins in the apoplast or the cytosol of host cells, resulting in effector-triggered plant susceptibility (ETS). Plants, in turn, have adapted to recognize these attacker-specific effectors by means of transmembrane or intracellular resistance proteins, triggering a superimposed layer of defense termed ETI (Jones and Dangl, 2006).

Both PTI and ETI are associated with the activation of a stereotypical set of physical and biochemical defense responses that are instrumental in halting pathogen ingress. These defenses include the biosynthesis of antimicrobial secondary metabolites, stomatal closure, bursts of reactive oxygen species, and local strengthening of plant cell walls by callose and lignin (Pieterse et al., 2009). Seminal experiments with mutant and transgenic plants in tobacco and *Arabidopsis* which are impaired in the biosynthesis, perception or signaling of specific hormones have demonstrated the central importance of plant hormones in the regulation of these downstream immune events. Upon pathogen attack, plants synthesize a complex blend of hormones, leading to the activation of distinct sets of defense-related genes (Glazebrook et al., 2003; Pieterse et al., 2012). It is thought that this signal signature, which differs considerably in timing, quantity, and composition according to the type of attacker encountered, plays a pivotal role in the regulation of the plant’s immune network and eventually determines the specific nature of the defense response triggered (De Vos et al., 2005; Mur et al., 2006).

## SALICYLIC ACID

Salicylic acid is a natural phenolic compound that plays well-known roles in the regulation of a wide variety of immune responses triggered by PAMPs and microbe-secreted effector proteins (Vlot et al., 2009; Boatwright and Pajerowska-Mukhtar, 2013). During PTI and ETI, endogenous levels of SA and its conjugates increase dramatically, preceding the induction of pathogenesis-related (PR) proteins and the onset of local and systemic acquired resistance (SAR; Metraux et al., 1990; Malamy and Klessig, 1992; Mishina and Zeier, 2007; Tsuda et al., 2009). SA biosynthesis in higher plants is derived from the shikimate-phenylpropanoid pathway, and may occur via two different branches. In *Arabidopsis*, basal SA production predominantly occurs via the conversion of phenylalanine to cinnamic acid by the enzyme phenylalanine ammonia lyase, whereas the majority of pathogen-induced SA production derives from isochorismate (Vlot et al., 2009).

In rice, however, the role of SA biosynthesis in disease resistance is still poorly understood, and even controversial. Driving the debate initially was the observation that rice accumulates high basal levels of SA (8–37 μg g^-1^ fresh weight) that do not change significantly upon pathogen attack (Silverman et al., 1995). In contrast, in tobacco and *Arabidopsis*, basal levels of SA are low (less than 100 ng g^-1^ fresh weight), but increase by two orders of magnitude following infection (Malamy and Klessig, 1992). Moreover, unlike dicots where *de novo* synthesized SA is rapidly converted into SA β-glucoside, in rice most SA is present in the free acid form (Silverman et al., 1995). Interestingly, these high levels of free SA are hypothesized to function as a preformed antioxidant, protecting rice plants from oxidative damage caused by aging, pathogen attack, or abiotic stress (Yang et al., 2004).

Despite its high endogenous SA content, rice is not insensitive to exogenously administered SA, but this is plant age-dependent. For instance, topical application of SA triggers resistance to the hemibiotrophic blast fungus *Magnaporthe oryzae* in adult plants but not in young seedlings (Iwai et al., 2007). Moreover, synthetic SA analogs such as probenazole, benzothiadiazole (BTH), and tiadinil induce defense responses in rice and, unlike in *Arabidopsis*, enhance resistance to a wide range of pathogens with different lifestyles and infection strategies, including *Magnaporthe oryzae*, the (hemi)biotrophic bacterial leaf blight pathogen *Xanthomonas oryzae* pv. *oryzae* (*Xoo*) and the necrotrophic root pathogens *Pythium graminicola* and *Hirschmanniella oryzae* (Shimono et al., 2007; De Vleesschauwer et al., 2008, 2012; Nahar et al., 2012; Xu et al., 2013). Considering that tiadinil and BTH act downstream of SA biosynthesis and that SA-deficient rice plants expressing the bacterial salicylate hydroxylase *NahG* display unaltered *PR* gene expression (Yang et al., 2004), these findings strongly suggest that the signaling action of SA, rather than its *de novo* biosynthesis, is an important factor mediating defense mobilization in rice.

Considerable differences between rice and *Arabidopsis* are also evident in the function of the upstream SA regulator phytoalexin deficient 4 (PAD4). *Arabidopsis PAD4* encodes a lipase-like protein that functions in ETI and basal immunity against biotrophic pathogens (Jirage et al., 1999; Lipka et al., 2005). AtPAD4 is also postulated to work in a positive feedback loop to promote SA biosynthesis in concert with another lipase-like protein, enhanced disease susceptibility 1 (AtEDS1; Zhou et al., 1998; Feys et al., 2001). Recent findings by the Wang lab, however, point to a very different function of PAD4 in rice (Ke et al., 2014). Unlike AtPAD4 which encodes a nucleocytoplasmic protein, OsPAD4 is situated in the plasma membrane. Moreover, OsPAD4-induced resistance to *Xoo* and the hemibiotrophic leaf streak pathogen *X. oryzae* pv. *oryzicola* (*Xoc*) does not involve SA-responses, but is associated with the accumulation of JA and the expression of JA-responsive genes.

Downstream of SA biosynthesis, the SA pathway in rice shares several signaling components with the SAR pathway in *Arabidopsis*, including the master regulatory protein NPR1. During SAR, SA-induced redox changes reduce the intermolecular disulphide bonds that normally keep NPR1 in an inactive oligomeric state in the cytosol (Tada et al., 2008). This reduction in turn releases monomeric NPR1, which is translocated to the nucleus where it interacts with TGA transcription factors (TFs) to activate defense gene expression (Zhang et al., 1999; Cheng et al., 2009). Recently, it was reported that NPR1 as well as its paralogs NPR3 and NPR4 may also serve as SA receptor proteins (Fu et al., 2012; Wu et al., 2012).

To date, five NPR1-like genes have been identified in the rice genome, among which OsNPR1 (also called OsNH1) is the closest homolog of AtNPR1 (Yuan et al., 2007). Ectopic expression of *OsNPR1* in rice induced constitutive accumulation of *PR* transcripts, conferring high levels of resistance to *Magnaporthe oryzae* and *Xoo* (Chern et al., 2005; Yuan et al., 2007; Sugano et al., 2010). By contrast, in rice and *Arabidopsis* overexpressing *AtNPR1*, defense-related genes are not activated until induced by abiotic stress, pathogen attack or BTH treatment (Cao et al., 1998; Fitzgerald et al., 2004).

Moreover, unlike the situation in *Arabidopsis* where nearly 99% of all BTH-responsive genes are controlled by NPR1 (Wang et al., 2006), many SA-responses in rice are controlled by a second master regulatory protein called OsWRKY45 which functions parallel to OsNPR1 (Shimono et al., 2007; Sugano et al., 2010). OsWRKY45 was originally identified as a BTH-responsive TF that is essential for resistance gene- and plant activator-mediated resistance to *Xoo* and *Magnaporthe oryzae* (Shimono et al., 2007, 2012; Inoue et al., 2013). Interestingly, OsWRKY45 also has been linked to stress responses in the endoplasmic reticulum (ER). Hayashi et al. (2012) showed that detection of ER stress by the transmembrane sensor protein IRE1 not only induces rapid expression of ER quality control-related chaperones, but also suppresses expression of *PR* genes and enhances transcription of *OsWRKY45* in an OsbZIP50-dependent manner. In contrast, concomitant activation of the SA response and ER stress induction suppresses the induction of ER quality control genes but promotes activation of OsWRKY45, which in turn triggers SA-responsive gene expression. Therefore, it is not unlikely that the SA response uses ER stress-induced OsWRKY45 to activate defense responses (Hayashi et al., 2012).

Although much remains to be discovered about the precise role of OsWRKY45 in the rice SA signaling pathway, several recent findings have provided new insights into the regulation of OsWRKY45. First, OsWRKY45 seems to be activated at least in part by an SA-dependent phosphorylation cascade controlled by the MAPKs OsMPK4 and OsMPK6 (Ueno et al., 2013). Although it is still unclear how this regulation affects OsWRKY45 activity, one interesting hypothesis is that phosphorylation of OsWKRY45 may facilitate its recruitment to the ubiquitin proteasome system (UPS). Like *Arabidopsis* NPR1 (Spoel et al., 2009), OsWRKY45 undergoes continuous degradation by the UPS in the nucleus (Matsushita et al., 2013). This UPS-mediated OsWRKY45 turnover likely plays a dual role by preventing spurious defense activation, on the one hand, and promoting the transcriptional activity of OsWRKY45 following SA treatment or pathogen attack, on the other (Matsushita et al., 2013). Interestingly, the same authors failed to show proteasome degradation of OsNPR1 under resting conditions (Matsushita et al., 2013). Whether OsNPR1 undergoes UPS-mediated turnover under pathogen challenge remains to be investigated.

Together, abovementioned findings highlight the unique complexities associated with SA signal transduction in rice. Most notably, the rice SA pathway seems to branch into two sub-pathways controlled by OsNPR1 and OsWRKY45. Recent microarray experiments revealed that almost half of all BTH-responsive genes and over two thirds of all BTH-downregulated genes are dependent on OsNPR1. These downregulated genes include many genes involved in photosynthesis and protein synthesis, suggesting a novel function of OsNPR1 in relocating energy and resources from house-keeping cellular activities to defense reactions (Sugano et al., 2010; Nakayama et al., 2013). In contrast, most genes upregulated by BTH are dependent on OsWRKY45, including many *PR* genes and a number of well-characterized defense-related TFs such as OsWRKY62, OsNAC4, and OsHSF1 (Takatsuji et al., 2010; Shimono et al., 2012; Nakayama et al., 2013). Together these findings favor a scenario whereby OsNPR1 and OsWRKY45 play different yet complementary roles in the rice SA pathway with OsNPR1 tentatively acting as an energy switch, enabling limited resources to be diverted to OsWRKY45-mediated pathogen defense.

## JASMONIC ACID

Jasmonic acid and its derivatives, collectively known as jasmonates, are lipid-derived hormones that regulate numerous physiological processes, including wound responses, secondary metabolite synthesis, and defense against biotic and abiotic stresses. In dicots, JA is widely believed to be predominantly effective against necrotrophic pathogens and herbivorous insects, whereas SA signaling is typically associated with immunity against biotrophs (Glazebrook, 2005). Although there is evidence for both positive and negative relationships between both pathways, the primary mode of interaction appears to be mutual antagonism, with corresponding trade-offs between biotrophs, on the one hand, and resistance to necrotrophs, on the other (Bostock, 2005). This SA–JA antagonism is evolutionary widely conserved and has been reported in as many as 17 plant species in various taxonomic groups (Thaler et al., 2012).

In rice, however, strikingly different results have been obtained with reports implicating JA in resistance against pathogens with distinct lifestyles and infection strategies. Perhaps most intriguingly, studies with JA-modified transgenics and pharmacological inhibitor experiments have uncovered JA as a powerful activator of resistance against the (hemi)biotrophic pathogens *Xoo* and *Magnaporthe oryzae* (Mei et al., 2006; Yamada et al., 2012; Riemann et al., 2013; Taniguchi et al., 2014). Although it could be argued that JA wards off these hemibiotrophs during the necrotrophic phase of their infection cycle, this is rather unlikely as *Xoo* has a very short necrotrophic stage while at least in some highly susceptible interactions *Magnaporthe oryzae* is thought to define a new paradigm for hemibiotropy in which each successive plant cell invasion is biotrophic, but individual invaded cells are no longer viable by the time the fungus moves into the next cell (Nino-Liu et al., 2006; Kankanala et al., 2007).

Consistent with its role in necrotroph resistance of dicots, JA is also effective against necrotrophic rice pathogens such as the sheath blight fungus *Rhizoctonia solani*. Taheri and Tarighi (2010) showed that topical JA application reduces sheath blight severity by almost 50% compared to non-treated controls. Enhanced sheath blight resistance concomitant with increased JA levels and induction of JA-responsive gene expression was also observed in rice plants overexpressing the pathogen-inducible TF gene *OsWRKY30* (Peng et al., 2012). In addition, the JA pathway is increasingly implicated in rice defenses against insect herbivores and root pathogens such as the biotrophic rice root knot nematode *Meloidogyne graminicola* (Zhou et al., 2009, 2014; Nahar et al., 2011; Tong et al., 2012; Ye et al., 2012). When considered together, these findings challenge the common assumption that JA triggers resistance to necrotrophs and susceptibility to biotrophs, and suggest that in rice there is no dichotomy between the effectiveness of the JA pathway and the lifestyle of the invading pathogen. Of particular note, it should be mentioned that although the role of SA in biotroph resistance and JA in necrotroph resistance is clear in many dicot pathosystems, there are also plenty of exceptions to this rule (Thaler et al., 2004; Glazebrook, 2005; Pieterse et al., 2009).

Despite the ability of JA to induce resistance against both (hemi)biotrophic and necrotrophic rice pathogens, several reports indicate that SA–JA antagonism is conserved in rice. In roots, SA attenuated JA-induced expression of the rice *PR* gene *RSOsPR10* and its negative regulator *OsERF1* (Takeuchi et al., 2011). Furthermore, in wounded rice plants, JA levels rise whereas SA levels decrease, suggesting negative crosstalk in the direction of JA damping SA action (Lee et al., 2004). Further evidence supporting antagonistic SA–JA signal interactions comes from gene expression experiments demonstrating enhanced transcript accumulation of the JA-responsive genes *OsAOS2* and *JaMYB* in SA-deficient *NahG* rice (Lee et al., 2001; Mei et al., 2006).

Over the past few years, various regulatory components involved in SA–JA crosstalk have been identified, key among which is NPR1 (Spoel et al., 2003; Pieterse et al., 2012; Thaler et al., 2012; Van der Does et al., 2013). Like its *Arabidopsis* counterpart, overexpression of *OsNPR1* is characterized by strong activation of SA-responsive genes and concomitant suppression of JA marker genes (Yuan et al., 2007). Moreover, similar to the situation in dicots, nuclear localization of OsNPR1 is required for SA-mediated defense gene expression, but not for suppression of JA signaling (Spoel et al., 2003; Yuan et al., 2007). *OsNPR1* antisense plants display elevated levels of JA and increased expression of JA biosynthetic genes upon insect infestation (Li et al., 2013). Accordingly, ectopic expression of *OsNPR1* not only confers enhanced resistance to *Magnaporthe oryzae* and *Xoo*, but also renders plants more susceptible to herbivorous insects. In a similar vein, rice plants overexpressing *AtNPR1* are hypersensitive to light (Fitzgerald et al., 2004) and display an increased susceptibility to viral infection and reduced tolerance to abiotic stress (Quilis et al., 2008). OsNPR1 thus seems to act as a positive regulator of SA-dependent pathogen resistance, while suppressing JA-mediated defenses to herbivorous insects and viral infection as well as tolerance to abiotic stresses.

A role in SA–JA crosstalk has also been suggested for OsWRKY13. Functioning upstream of OsNPR1 and OsWRKY45, this TF positively regulates SA-mediated rice defenses while suppressing the JA pathway (Qiu et al., 2007, 2008, 2009; Tao et al., 2009). Recently, OsWRKY13 was also shown to repress drought tolerance by transcriptionally suppressing the downstream TF SNAC1 (Xiao et al., 2013).

Although abovementioned studies clearly indicate the potential for negative SA–JA signal crosstalk, examples of positive interactions have also been reported, both in dicot and monocot systems (Mur et al., 2006; Pieterse et al., 2009). In general, however, positive SA–JA signal interactions appear to be more common in rice than in dicots. Most tellingly in this regard, recent microarray studies showed that unlike in dicots more than half of all BTH- or SA-upregulated rice genes are also induced by JA (Garg et al., 2012; Tamaoki et al., 2013). Moreover, similar to the *Arabidopsis cpr5*, *cpr6*, *cpr22*, *cet,* and *hrl1* mutants (Clarke et al., 1998, 2000, 2009; Hilpert et al., 2001; Yoshioka et al., 2001; Devadas et al., 2002), several rice mutant and transgenic lines display simultaneously elevated SA and JA signaling. For instance, rice plants mutated in the hydroperoxide lyase *OsHPL3* display strongly enhanced JA levels concomitant with increases in SA production and heightened expression of SA-responsive *PR* genes (Liu et al., 2012b; Tong et al., 2012). Activation of JA synthesis was also found to prime herbivore-induced SA synthesis in rice plants silenced for the phospholipase D genes *OsPLDα3* and *OsPLDα4* (Qi et al., 2011), while Pan et al. (2014) reported increased expression of both SA- and JA-biosynthesis genes in sheath blight-resistant rice lines overexpressing the JA and ET-inducible TF JERF1. Together, these findings bring a new twist to the classical crosstalk model and suggest that although hyperactivation of one has the ability to override the other, rice SA and JA pathways may feed into a common defense system that is effective against different types of attackers. In support of this concept, it was previously suggested that SA and JA act synergistically when applied at low concentrations, whereas a high concentration of one hormone antagonizes the other (Mur et al., 2006).

## ETHYLENE

Ethylene is a small gaseous hormone that controls diverse aspects of plant life. In plant immunity, ET is generally thought to act in concert with JA to induce necrotroph resistance while antagonizing SA-mediated biotroph resistance (Derksen et al., 2013). Accumulating evidence, however, indicates that ET can interact both positively and negatively with SA, depending on the infection strategy of the invading pathogen (van der Ent and Pieterse, 2012; Derksen et al., 2013). Like SA and JA, ET is rapidly synthesized following PAMP perception (Boller and Felix, 2009; Schwessinger and Ronald, 2012). Although the precise function of ET in PTI is still elusive, recent evidence suggest a combined role of ET and endogenous peptides in an amplification loop required for sustained PTI (Liu et al., 2013b; Tintor et al., 2013).

In rice, accumulation of ET and its coproduct cyanide was found to be indispensable ETI against the hemibiotroph *Magnaporthe oryzae* (Iwai et al., 2007). Interestingly, activation of ET synthesis was also shown to be responsible for the partial blast resistance of rice plants growing in anaerobic conditions such as moisture-saturated soils or flooded paddies (Singh et al., 2004). Transgenic lines overexpressing *OsACS2*, a gene encoding the key ET biosynthesis enzyme 1-aminocyclopropane-1-carboxylic acid synthase (ACS), showed increased resistance to both *Magnaporthe oryzae* and *R. solani*, whereas silencing of ET biosynthesis genes or the central ET signal transducer OsEIN2b rendered plants more susceptible against *Magnaporthe oryzae* and the bacterial pathogen *Burkholderia glumae* (Bailey et al., 2009; Seo et al., 2011).

Abovementioned studies clearly indicate that ET plays an important role in rice defense to various pathogens. However, as in dicots, ET can also act negatively on rice immunity, as was shown for the necrotrophic rice brown spot fungus *Cochliobolus miyabeanus*. Exogenously applied Ethephon (which is converted to ET in plant cells) strongly promotes disease development in this interaction, whereas genetic or pharmacological disruption of ET signaling resulted in enhanced resistance (De Vleesschauwer et al., 2010). Moreover, gene expression experiments revealed a strong activation of ET signaling in susceptible but not in resistant rice plants. Although preliminary, these findings strongly suggest that *C. miyabeanus* exploits ET as virulence factor and co-opts the rice ET signaling route to rewire the rice signaling circuitry and antagonize host immunity.

A negative impact of ET on rice disease resistance has also been observed for *Xoo*. Shen et al. (2011) reported that silencing of the MAPKKK OsEDR1 resulted in reduced expression of *ACS* genes, low levels of ET, and enhanced resistance to *Xoo*. Interestingly, this resistance was accompanied with increased SA and JA synthesis and constitutive expression of SA- and JA-marker genes, suggesting that when ET is lowered, levels of SA and JA increase (Shen et al., 2011). Together these observations suggest that ET plays a complex and ambiguous role in the rice immune system, the effect of which may depend not only on the lifestyle and overall infection biology of the attacking pathogen, but also on specialized features of each interaction. In compliance with this concept, Lu et al. (2014) recently also reported a contrasting role of ET biosynthesis in resistance of rice against chewing and phloem-feeding insects.

## ABSCISIC ACID

Compared with the classic defensive hormones SA, JA, and ET, the role of the ‘abiotic stress hormone’ ABA in regulating plant immunity is much less understood, and even controversial. Recent studies in dicots showed divergent and complex effects of ABA on defense responses, including the suppression of SA- and JA/ET-dependent defenses, synergistic crosstalk with JA signaling, suppression of ROS generation, induction of stomatal closure, and stimulation of callose deposition (Asselbergh et al., 2008; Cao et al., 2011). In general, the impact of ABA on plant defense seem to be plant–pathogen interaction-specific, rather than to rely on the lifestyle or infection strategy of the pathogen. In *Arabidopsis*, for instance, ABA both positively and negatively regulates resistance to the necrotrophic fungi *Alternaria brassicicola* and *Botrytis cinerea*, respectively (Adie et al., 2007). The timing of infection and type of tissue are other crucial factors underlying ABA modulation of plant immunity. This is nicely exemplified in *Pseudomonas syringae* pv. *tomato*-infected *Arabidopsis* where ABA prevents pathogen entry by inducing stomatal closure, yet is hijacked by the same pathogen to antagonize post-invasive disease resistance by suppressing SA-dependent defenses in the apoplast (Melotto et al., 2006; Mohr and Cahill, 2007; de Torres-Zabala et al., 2009).

In common with these findings, ABA also plays ambiguous roles in the rice defense-signaling network. We previously showed that exogenous ABA enhances basal resistance of rice against the necrotroph *C. miyabeanus* (De Vleesschauwer et al., 2010). This ABA-inducible resistance was associated with restriction of fungal progression in the mesophyll and was dependent on negative crosstalk with the rice ET-signaling pathway (De Vleesschauwer et al., 2010). In a similar manner, it has been proposed that ABA conditions susceptibility to both *Magnaporthe oryzae* and *Xoo* by antagonizing effectual SA-mediated defenses upstream or at the level of OsNPR1 and OsWRKY45 (Jiang et al., 2010; Xu et al., 2013). Interestingly, infection by *Magnaporthe oryzae* and *Xoo* is tightly associated with greatly elevated ABA levels and extensive reprogramming of ABA-responsive genes (Ribot et al., 2008; Liu et al., 2012a; Xu et al., 2013). Consistent with previous findings in the *Arabidopsis*–*Pseudomonas syringae* pv. *tomato* pathosystem (de Torres-Zabala et al., 2007, 2009; Goel et al., 2008; Ho et al., 2013), it therefore appears that both pathogens may hijack the rice ABA pathway to cause disease. In support of this assumption, *Magnaporthe oryzae* was recently shown to produce and secrete ABA *in vitro* and *in planta* (Jiang et al., 2010). Since ABA has no apparent impact on the pathogen’s physiology, one may hypothesize that *Magnaporthe oryzae* uses its own ABA to activate ABA signaling in host cells, thereby suppressing the SA- and ET-signaling pathways that normally serve to limit pathogen growth (Takatsuji and Jiang, 2014).

Although understanding of molecular components governing signal transduction and sensitivity in the rice ABA signaling network is still in its infancy, accumulating evidence points toward a crucial role of the ABA-inducible protein kinase OsMPK5. OsMPK5 RNAi lines show increased levels of ET and enhanced resistance to multiple hemibiotrophic pathogens including *Magnaporthe oryzae*, *Xoo*, *Burkholderia glumae* and the migratory nematode *Hirschmanniella oryzae* (Xiong and Yang, 2003; Nahar et al., 2012; Xu et al., 2013); however, they are also impaired in ABA-inducible resistance to *C. miyabeanus* and are hypersensitive to abiotic stresses (Bailey et al., 2009; De Vleesschauwer et al., 2010). Conversely, silencing of the central ET-signal transducer *OsEIN2* resulted in enhanced resistance to *C. miyabeanus* as well as hypersensitivity to *Magnaporthe oryzae*, *Xoo*, ABA and abiotic stress (Bailey et al., 2009; De Vleesschauwer et al., 2010). Together, these findings suggest that OsMPK5 and OsEIN2 act as molecular switches between the rice ABA and ET pathways, thereby differentially regulating abiotic stress tolerance and *C. miyabeanus* resistance, on the one hand, and defense against hemibiotrophic pathogens, on the other.

Interestingly, OsMPK5 also positively interacts with the JA pathway in protecting rice against chewing herbivores, suggesting positive crosstalk in the direction of ABA boosting JA action (Wang et al., 2013). However, Nahar et al. (2013) reported that ABA disables JA-induced resistance against *Hirschmanniella oryzae*, indicating that the nature of interaction between these pathways is complex and also attacker dependent.

## NEW KIDS ON THE BLOCK: DEVELOPMENTAL HORMONES DO DEFENSE

Contrary to the classic defense hormones SA, JA, and ET and the ‘abiotic stress hormone’ ABA, other hormones including auxins, gibberellins, brassinosteroids (BRs), and CKs, were historically best studied for their role in growth and development and only recently emerged as additional players in plant–microbe interactions. Although their precise role and function in orchestrating plant defense is still elusive, recent data are now beginning to unveil how these ‘developmental’ hormones modulate host immunity, and how microbe-induced perturbations of these classic growth regulators contribute to virulence.

### AUXINS

Auxins, such as indole-3-acetic acid (IAA), are a major class of plant hormones that control a range of cellular processes, including apical dominance, tropistic growth, lateral root formation, vascular tissue development, and regulation of plant senescence. Thus far, studies on *Arabidopsis* imply that auxin attenuates (hemi)biotroph resistance but enhances plant defenses toward necrotrophic pathogens (Fu and Wang, 2011). In compliance with this concept, auxin and more specifically IAA, also act as virulence factors of the hemibiotrophic rice pathogens *Magnaporthe oryzae*, *Xoo* and *Xoc,* causal agent of bacterial leaf streak disease (Ding et al., 2008; Domingo et al., 2009; Fu et al., 2011). Like many other microbes, these pathogens produce and secrete IAA themselves and also increase IAA biosynthesis and signaling upon infection (Fu et al., 2011). In plants, auxin levels are regulated in part through negative feedback by a group of auxin-inducible GH3 (Gretchen Hagen 3) family genes that catalyze the conjugation of IAA to various amino acids. Unlike in *Arabidopsis* where three distinct groups of GH3 enzymes have been identified, only groups I and II are present in rice (Westfall et al., 2010). Up to now, three group II GH3 enzymes have been functionally characterized in rice, namely OsGH3.1, OsGH3.2, and OsGH3.8. Consistent with IAA promoting hemibiotroph susceptibility, rice transformants overexpressing these enzymes displayed reduced levels of IAA and enhanced resistance to *Magnaporthe oryzae*, *Xoo* and *Xoc* (Ding et al., 2008; Domingo et al., 2009; Fu et al., 2011).

In *Arabidopsis*, auxin is widely believed to antagonize SA-mediated defenses against biotrophic pathogens (Zhang et al., 2007; Truman et al., 2010). Two recent papers, however, suggest that auxin can also promote growth of *Pseudomonas syringae* pv. *tomato* and disease development in *Arabidopsis* via a mechanism independent of suppression of SA action (González-Lamothe et al., 2012; Mutka et al., 2013). These findings echoe previous studies in rice where IAA-induced hemibiotroph susceptibility was found to be independent of SA and JA (Ding et al., 2008; Fu et al., 2011). Instead, it has been proposed that pathogen-triggered IAA promotes susceptibility by inducing the expression of cell wall-loosening expansions, thereby facilitating pathogen entry and allowing increased nutrient leakage. Notably, *OsGH3.2*-overexpressing plants not only exhibit decreased IAA levels, but also produce less ABA, which may contribute to the resistance against *Magnaporthe oryzae* and *Xoo* (Du et al., 2012). In addition, these plants are also more tolerant to cold and oxidative stresses, providing a genetic strategy for breeding rice with broad-spectrum stress tolerance using *GH3* family genes. In this context, it will be particularly interesting to assess whether auxin promotes resistance to necrotrophic rice pathogens, as was previously shown in *Arabidopsis* (Llorente et al., 2008).

### CYTOKININS

Cytokinins are a group of *N^6^*-substituted adenine derivatives that orchestrate myriad growth and developmental processes in plants. As one of the latest hormones to be linked with immunity, the precise role of CKs in plant immunity remains to be fully elucidated. Historically, CKs are associated with disease symptoms and morphological anomalies, such as fasciation, senescence, and the formation of galls, tumors and so-called ‘green islands’ (Grant and Jones, 2009). Many fungal and bacterial pathogens can produce CK themselves and/or increase CK synthesis in plants (Siemens et al., 2006; Walters et al., 2008; Naseem et al., 2014). Moreover, pathogens may also activate plant CK signaling in order to suppress host immunity. For instance, it was recently shown that *Pseudomonas syringae* pv. *tomato* deploys the effector HopQ1 to activate plant CK signaling and suppress PTI via down-regulation of the flagellin receptor gene *FLS2* (Hann et al., 2014).

Although these observations indicate a role of CKs in promoting pathogen virulence, recent work has revealed that CKs can also augment plant immunity against a fairly broad range of pathogens exhibiting different lifestyles (Swartzberg et al., 2008; Grosskinsky et al., 2011; Argueso et al., 2012; Naseem et al., 2012, 2014). Argueso et al. (2012) argued that the levels of CK are important in determining the amplitude of plant immunity. In this study, low concentrations of the CK benzyl adenine (BA) promoted susceptibility of *Arabidopsis* to the biotroph *Hyaloperonospora arabidopsidis*, whereas high BA concentrations enhanced disease resistance by priming the SA defense pathway. Biochemical analyses revealed that this positive CK–SA crosstalk is mediated through a direct interaction between the CK-activated TF ARR2 and the SA response factor TGA3, resulting in potentiated promoter binding of TGA3 and increased expression of SA-dependent defense genes (Choi et al., 2010, 2011). Meanwhile, SA feedback-inhibits CK signaling, which may serve to fine-tune the effect of CK in plant immunity (Argueso et al., 2012). In tobacco, however, a different mechanism appears to be operative. In this plant species, CK enhances resistance to *Pseudomonas syringae* pv. *tobacco* independently of SA, indicating that nuanced, species-specific mechanisms underlie CK’s immune-regulatory function (Grosskinsky et al., 2011).

Although exogenous application of CK at low and high concentrations did not alter *Magnaporthe oryzae* progression in rice, CK was found to synergistically interact with SA to activate *PR* genes in detached leaf assays (Jiang et al., 2013). A follow-up study by the same authors revealed that CK also joins forces with SA to trigger the production of diterpenoid-type phytoalexins in an OsWRKY45-dependent manner (Akagi et al., 2014). Paradoxically, *Magnaporthe oryzae* secretes CK itself and activates CK signaling in infected leaves, which may facilitate blast infection by increasing the sink strength of infected tissues (Jiang et al., 2013).

### BRASSINOSTEROIDS

Brassinosteroids are a unique group of plant steroidal hormones that play pivotal roles in cell expansion and division, differentiation and reproductive development. Although BRs have long been seen as mainly positive players in plant immunity, recent findings in both dicots and rice suggest a more complex situation, with positive, negative as well as neutral BR effects being reported that are seemingly independent of either the plant species or type of pathogen involved (De Bruyne et al., 2014). In rice, for instance, BR promotes resistance to the hemibiotrophic leaf pathogens *Xoo* and *Magnaporthe oryzae*, while inducing susceptibility to the hemibiotrophic root-knot nematode *Meloidogyne graminicola* and the necrotrophic oomycete *Pythium graminicola* (Nakashita et al., 2003; De Vleesschauwer et al., 2012; Nahar et al., 2013).

Accordingly, recent studies have revealed a wide variety of underpinning mechanisms, ranging from orchestration of oxidative metabolism and secondary metabolite production to modulation of PAMP perception and ensuing PTI signaling (De Bruyne et al., 2014). Depending on among others the relative hormone concentration and their effect on the BR co-receptor BAK1, BRs can act both antagonistically and synergistically with PTI responses (Albrecht et al., 2012; Belkhadir et al., 2012; Lozano-Durán et al., 2013; Shi et al., 2013). In addition, BRs have been found to cross-communicate with a range of other hormones, including SA, JA, ABA, auxins, and gibberellins (De Bruyne et al., 2014). Consistent with its apparent pluriform role in regulating plant immune responses, the nature and direction of this BR hormone crosstalk can vary widely. For instance, whereas there is evidence for synergistic BR–SA crosstalk in *Arabidopsis*, previous studies with rice revealed that BR enhances resistance to *Xoo* and *Magnaporthe oryzae* in an SA-independent manner while disabling SA-mediated defenses against root pathogens (Nakashita et al., 2003; Divi et al., 2010; De Vleesschauwer et al., 2012). Much like gibberellins and CKs, BRs thus seem to play ambiguous roles in the plant defense network, the effect of which may depend not only on the pathogen’s lifestyle and infection strategy, but also on spatial and temporal conditions.

### GIBBERELLINS AND DELLA PROTEINS

Gibberellins are a class of tetracyclic diterpenoid hormones that affect nearly every aspect of plant growth and development. According to current concepts, GAs promotes plant growth by inducing the degradation of a class of nuclear proteins, called DELLAs. *Arabidopsis* mutants lacking four of the five DELLA proteins showed attenuated induction of the JA marker gene *Pdf1.2*, resulting in enhanced susceptibility to the necrotrophic fungus *Alternaria brassicicola* (Navarro et al., 2008). In contrast, the same mutants exhibited increased levels of resistance to the hemibiotrophic bacterium *Pseudomonas syringae* pv. *tomato* accompanied with elevated levels of SA (Navarro et al., 2008). On the basis of these and other findings, it was proposed that DELLAs modulate the strength of SA/JA signaling during plant immunity, promoting JA perception and/or signaling, and repressing SA biosynthesis and signaling. Accordingly, pretreatment with GA restricts JA signaling, resulting in enhanced SA signaling and increased biotroph resistance (Navarro et al., 2008).

In rice, however, strikingly different results have been obtained in that exogenous GA was found to enhance susceptibility against the hemibiotrophic pathogens *Xoo* and *Magnaporthe oryzae* (Yang et al., 2008). Moreover, ectopic expression of a GA-deactivating enzyme designated elongated uppermost internode (EUI) significantly reduced rice SA and JA levels and enhanced resistance to the latter pathogens, whereas EUI loss-of-function mutations led to increased susceptibility (Yang et al., 2008). Other mutants deficient in biosynthesis or perception of GA showed similar gain-of-resistance phenotypes when challenged with either *Xoo* or *Magnaporthe oryzae* (Tanaka et al., 2006; De Vleesschauwer et al., 2013; Qin et al., 2013). On the other hand, GA was shown to be a positive player in resistance against the necrotrophic root pathogen *Pythium graminicola* (De Vleesschauwer et al., 2012). Therefore, opposite to the situation in *Arabidopsis*, rice GA signaling appears to induce susceptibility to hemibiotrophic pathogens and resistance to necrotrophs.

Although much remains to be discovered about the precise mechanisms via which GA and DELLAs modulate plant immunity, recent studies have implicated DELLAs in a variety of processes, including the regulation of oxidative and energy metabolism, cell wall development, and cytoskeleton architecture (De Bruyne et al., 2014). Moreover, evidence is accumulating that DELLAs orchestrate plant immunity via competitive binding to JA ZIM-domain (JAZ) proteins, a family of JA signaling repressors. JAZ proteins bind and inhibit the activity of numerous TFs, including the key JA transcriptional activator MYC2 (Kazan and Manners, 2012, 2013). Recently, three groups have shown that DELLAs compete with MYC2 for binding to JAZs, thereby releasing free MYC2 to activate JA-responsive gene expression and, hence, increase resistance to necrotrophic pathogens (Hou et al., 2010; Wild et al., 2012; Yang et al., 2012). In the presence of GA, however, DELLAs are rapidly degraded, leading to inhibitory JAZ-MYC2 interactions and disruption of JA signaling. This so-called ‘relief of repression’ model not only elegantly explains how plants balance growth and defense responses, but also offers novel insights into how GA disables JA-mediated necrotroph resistance by degrading DELLAs and releasing JAZs to bind and inhibit MYC2.

Consistent with these findings in *Arabidopsis*, Yang et al. (2012) demonstrated that SLR1, the only DELLA in rice, serves as a main target of JA-mediated growth inhibition and is required for full-scale activation of JA-responsive gene expression in rice. In turn, JA treatment protects SLR1 from degradation by exogenously administered GA, suggesting reciprocal synergism between the JA and SLR1 signaling pathways (Yang et al., 2012). Considering that many other hormones affect DELLA protein stability, either directly or indirectly, it thus seems that SLR1 is positioned at the intersection of various hormone pathways, acting as a main hub for signal crosstalk and pathway integration (De Bruyne et al., 2014).

## TIME FOR ACTION: TOWARD ENGINEERING HORMONE-BASED SUSTAINABLE DISEASE RESISTANCE IN RICE

The past decade has seen tremendous progress in understanding hormone perception and signaling in rice and its role in molding pathological outcomes. Although far from complete, our knowledge of the rice defense signaling network may now be detailed enough to contemplate the rational deployment of it for engineering disease-resistant rice plants.

To date, most translational efforts have focused on reinforcing rice defense signaling by transgenic manipulation of hormone signaling components. Given its strong impact on resistance against the hemibiotrophs *Magnaporthe oryzae* and *Xoo* and its ability to reduce susceptibility against the necrotrophic root pathogens *Pythium graminicola* and *Hirschmanniella oryzae*, SA regulatory elements are ideal candidates for engineering broad-spectrum disease resistance in rice. Unfortunately, the disease resistance governed by constitutive overexpression or knockout of positive and negative regulators of the SA pathway, respectively, is most often accompanied by severe growth defects and/or impaired tolerance to abiotic stresses. Thus, various groups have shown that high-level expression of *OsNPR1* and *OsWRKY45* results in strong growth retardation and formation of lesion-mimics, while transgenic rice lines overexpressing *OsWRKY13* are hypersensitive to drought (Chern et al., 2005; Shimono et al., 2007; Xiao et al., 2013). These negative effects, commonly referred to as trade-offs, are attributed to the reallocation of resources from growth to defense against the most life-threatening stress and must be overcome to maximize rice yield under variable environmental conditions (Sharma et al., 2013; Huot et al., 2014).

One approach to bypass trade-offs is by managing the expression level of the transgene. Recent findings revealed that moderate expression of *OsWRKY45* under control of a weak constitutive promoter largely eliminated the fitness costs and environmental sensitivity related to strong *OsWRKY45* expression, while retaining disease resistance (Takatsuji and Jiang, 2014). Similarly, low-level silencing of *OsSSI2*, a negative regulator of the rice SA pathway, resulted in strong resistance to *Magnaporthe oryzae* and *Xoo* without appreciable growth defects, whereas *OsSSI2* knockout plants were severely stunted (Jiang et al., 2009). The use of highly specific pathogen-inducible promoters is an additional strategy to fine-tune the location, timing and magnitude of transgene expression. To avoid spurious defense activation, ideal candidate promoters are those that are not PAMP responsive but are rapidly induced by virulent pathogens (Grant et al., 2013). Although relatively few effector-responsive rice genes have been identified thus far, the increasing availability of high temporal microarray data of a range of rice-pathogen interactions may open the door to identifying tightly controlled transcriptional units that can be used to drive transgene expression when and where pathogens invade.

In addition, trade-offs may be circumvented by engineering transgenic rice lines that are designed to neutralize microbial hormone intervention strategies. Such proactive response necessitates the identification and precision engineering of core pathogen-modulated hormone signaling elements and re-wiring these components to promoters specifying precise, temporally and spatially controlled responses to pathogens (Grant et al., 2013). Interfering with hormone-based virulence strategies seems especially promising for pathogens such as *Xoo* and *Magnaporthe oryzae* which are thought to hijack the rice ABA, auxin, and GA pathways to induce host susceptibility (see above).

To prevent microbial manipulation of these pathways, one could envisage creating a dominant hormone-insensitive phenotype, either via local and timely disruption of hormone perception or through ectopic expression of re-engineered negative regulators of hormone signaling such as ABA-repressing Clade A protein phosphatase 2Cs (PP2C). Upon ABA binding, a family of cytosolic ABA receptors commonly known as PYLs (PYRABACTIN RESISTANCE LIKE) bind to and inhibit the active site of PP2Cs. This alleviates negative regulation on the PP2C target sucrose non-fermenting related kinase 2 (SnRK2), leading to activation of ABA signaling (Cutler et al., 2010; Weiner et al., 2010). Interestingly, mutations of specific residues residing in the active site of PP2Cs disrupt PYL-PP2C interactions but retain the ability to dephosphorylate target SnRK2s (Miyazono et al., 2009; Melcher et al., 2010). Driving the expression of such mutated PP2Cs may thus inactivate ABA signaling at two key nodes (Grant et al., 2013).

A similar strategy could be followed for SLR1 and Aux/IAA proteins, which function as the central repressors of GA and auxin signaling, respectively. Upon hormone perception, these negative regulators are targeted by cognate F-box proteins for polyubiquitination and subsequently degraded by the 26S proteasome, thus relieving their repressive effect. Structural and predictive modeling have revealed the domains and residues involved in SLR1 and Aux/IAA protein–protein interactions (Gray et al., 2001; Hirano et al., 2010; Sato et al., 2014). Therefore, one may consider re-wiring engineered SLR1 and Aux/IAA variants that no longer bind the corresponding F-box proteins GID2 and TIR1 and, hence, are resistant to UPS-mediated degradation. In a dual approach that complements the re-wiring of hormone signaling pathways, it would also be possible to alleviate pathogen-induced hormone accumulation by connecting well-characterized hormone catabolism genes such as ABA 8′-hydroxylases, GA 2-oxidases or auxin-inducible GH3 enzymes to pathogen-responsive promoters (Saika et al., 2007; Lo et al., 2008; Jain and Khurana, 2009).

## CONCLUDING REMARKS

Over the past decade, significant inroads have been made in our understanding of hormone defense signaling in *Arabidopsis* and other dicot plants. However, as illustrated throughout this review, the conceptual framework emerging from these studies does not always translate to monocot systems (**Table 1**). While underscoring the importance of using alternative models systems, the unique complexities associated with defense networking in rice call for a re-evaluation of overly generalized defense models.

**Table 1:** Differences and commonalities in hormone defense networking in rice and *Arabidopsis*.

Hormone	*Arabidopsis thaliana*	*Oryza sativa*
Salicylic acid	– Effective mainly against biotrophs	– Effective against (hemi)biotrophs and necrotrophs
	– Low basal levels of SA, strong rise upon pathogen attack	– High basal levels of SA that do not change upon pathogen attack and likely act as a preformed antioxidant
	– SA signaling is controlled by the master regulator NPR1	– 2-branched signaling pathway controlled by the master regulators NPR1 and WRKY45
	– NPR1 function requires degradation by the ubiquitin proteasome system (UPS)	– WRKY45 function requires degradation by the UPS, NPR1 is not degraded by the UPS under resting conditions
	– NPR1 antagonizes JA-responsive gene expression	– NPR1 antagonizes JA-responsive gene expression
Jasmonic acid	– Effective mainly against necrotrophs	– Effective against (hemi)biotrophs and necrotrophs
	– Negative interactions with SA pathway prevail	– Positive interactions with SA pathway prevail
Ethylene	– Suppresses SA-dependent biotroph resistance	– Variable effects on plant immunity independent of the pathogen’s lifestyle
	– Co-operates with JA to promote resistance against necrotrophs	
Auxin	– Suppresses SA-dependent biotroph resistance	– Suppresses resistance to (hemi)biotrophs independently of SA and JA
	– Promotes JA-dependent necrotroph resistance	– Effect against necrotrophs unknown
Gibberellic acid	– Five DELLA proteins: RGA, GAI, RGL1, RGL2, and RGL3	– A single DELLA protein: SLR1
	– DELLAs interact with JAZs to promote JA-dependent necrotroph resistance	– SLR1 promotes JA-dependent resistance against (hemi)biotrophs
	– DELLAs suppress SA-dependent biotroph resistance	
Cytokinin	– Promotes SA-responsive gene expression	– Promotes SA-responsive gene expression
	– Variable effects on immunity independent of the pathogen’s lifestyle	– Variable effects on immunity independent of the pathogen’s lifestyle
Brassinosteroids	– Variable effects on immunity independent of the pathogen’s lifestyle	– Variable effects on immunity independent of the pathogen’s lifestyle
Abscisic acid	– Variable effects on immunity independent of the pathogen’s lifestyle	– Variable effects on immunity independent of the pathogen’s lifestyle

**FIGURE 1:**
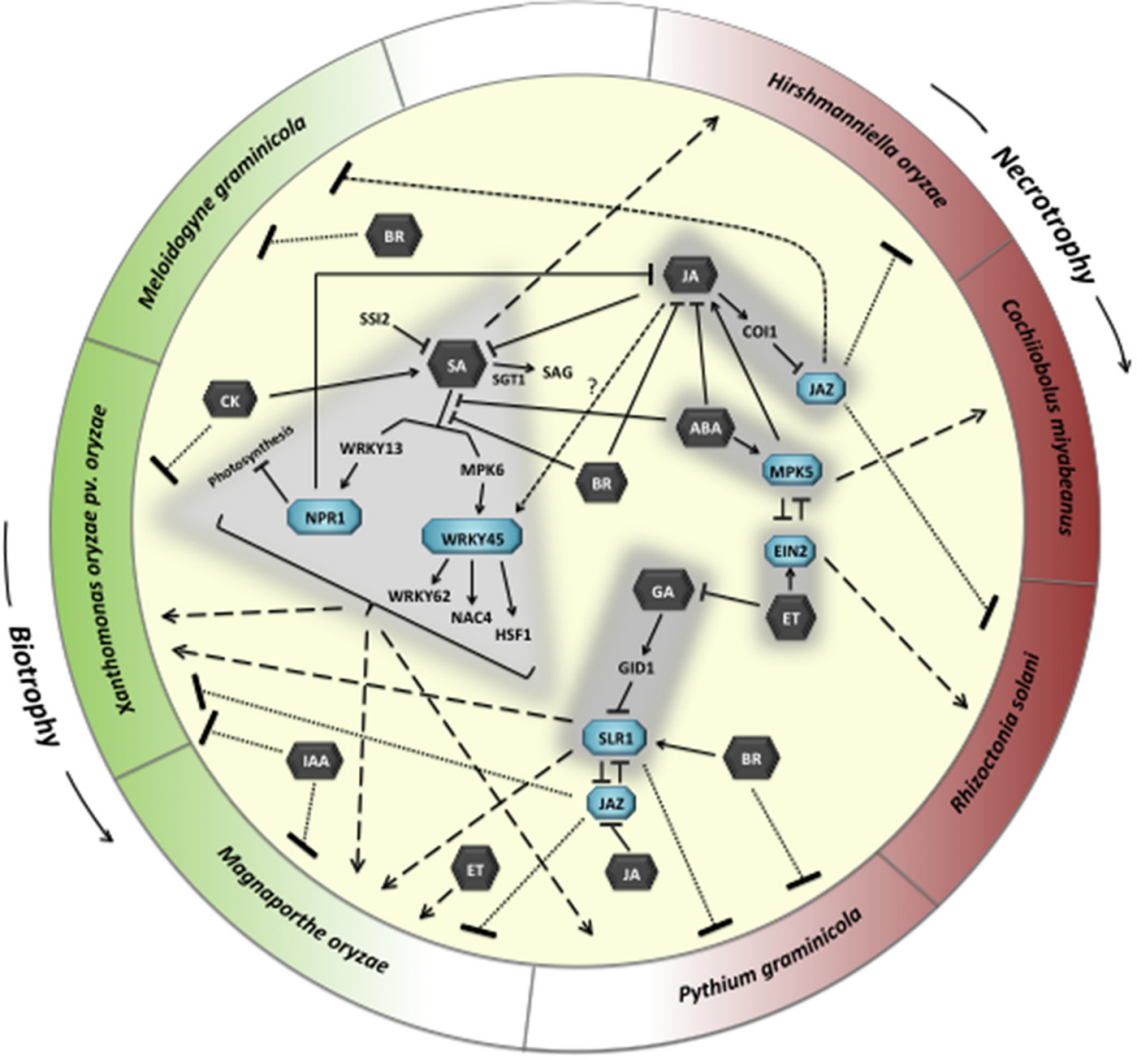
**Model illustrating the role of hormone signaling pathways and their crosstalk in molding disease outcomes in rice.** Sharp arrows indicate positive effects, blunt-ended lines depict negative effects. SA, salicylic acid; JA, jasmonic acid; ET, ethylene; ABA, abscisic acid; CK, cytokinin; IAA, auxin; GA, gibberellic acid; BR, brassinosteroids.

Contrary to the classic binary defense model with SA and JA playing opposite roles in biotroph and necrotroph resistance, respectively, innate immunity in rice appears to be controlled by a much more complicated signaling network that supports no clear dichotomy between the effectiveness of most hormone pathways and the overall infection biology of the invading pathogen (**Figure 1**). Most conspicuously, although hyperactivation of one can attenuate the other, synergistic SA–JA interactions seem to prevail in rice and the two hormones are effective against both hemibiotrophic and necrotrophic rice pathogens. Moreover, unlike in dicots, we are unaware of any reports showing negative effects of SA or JA on rice pathogen defenses. Therefore, it is not inconceivable that both hormones function as endogenous priming agents that amplify infection-induced defense reactions regardless of the lifestyle of the invading pathogen. In contrast, ET can have both positive and negative effects on rice disease resistance that are seemingly independent of the pathogen’s parasitic habits.

The impact of developmental hormones is equally complex. As was reported in *Arabidopsis*, auxin promotes susceptibility to (hemi)biotrophic rice pathogens, while CK signaling can cascade either to the detriment or the benefit of plant. GAs, on the other hand, appear to play opposite roles in rice and *Arabidopsis*. Finally and consistent with their ambivalent role in dicot immunity, BRs and ABA can both promote and suppress rice immunity depending not only on the type of pathogen, but also on the type of tissue, and even spatial and temporal conditions.

Despite the recent progress, much remains to be learned about the role of hormones in the regulation of the rice defense signaling network. For instance, it is still unclear how SA and JA are perceived in rice and how their signaling pathways interact at the molecular level, there is little information available about the impact of viruses, insects and nematodes on the rice hormone signaling network, and there is still much to be learned about the hormone intervention strategies used by rice pathogens to inflict disease. Moreover, few studies have investigated the spatiotemporal dynamics of a given hormone during rice-pathogen interactions and none addresses the kinetics and signature of the blend of hormones released upon pathogen attack. Finally, there is a paucity of knowledge on the molecular players orchestrating pathway crosstalk and signal integration in the rice signaling circuitry. Deepening our knowledge in this area is especially important since defining synergies and trade-offs may help identify appropriate contexts for the optimal deployment and commercial acceptance of hormone-based rice disease resistance.

## Conflict of Interest Statement

The authors declare that the research was conducted in the absence of any commercial or financial relationships that could be construed as a potential conflict of interest.
